# The GlyR Extracellular β8–β9 Loop – A Functional Determinant of Agonist Potency

**DOI:** 10.3389/fnmol.2017.00322

**Published:** 2017-10-09

**Authors:** Dieter Janzen, Natascha Schaefer, Carolyn Delto, Hermann Schindelin, Carmen Villmann

**Affiliations:** ^1^Institute for Clinical Neurobiology, University of Würzburg, Würzburg, Germany; ^2^Rudolf Virchow Center for Experimental Biomedicine, University of Würzburg, Würzburg, Germany

**Keywords:** glycine receptor, β8–β9 loop, side chain length, side chain volume, ligand potency, gating, startle disease

## Abstract

Ligand-binding of Cys-loop receptors results in rearrangements of extracellular loop structures which are further translated into the tilting of membrane spanning helices, and finally opening of the ion channels. The cryo-EM structure of the homopentameric α1 glycine receptor (GlyR) demonstrated an involvement of the extracellular β8–β9 loop in the transition from ligand-bound receptors to the open channel state. Recently, we identified a functional role of the β8–β9 loop in a novel startle disease mouse model *shaky*. The mutation of residue GlyRα1^Q177^ to lysine present in *shaky* mice resulted in reduced glycine potency, reduced synaptic expression, and a disrupted hydrogen network at the structural level around position GlyRα1^Q177^. Here, we investigated the role of amino acid volume, side chain length, and charge at position Q177 to get deeper insights into the functional role of the β8–β9 loop. We used a combined approach of *in vitro* expression analysis, functional electrophysiological recordings, and GlyR modeling to describe the role of Q177 for GlyR ion channel function. GlyRα1^Q177^ variants do not disturb ion channel transport to the cellular surface of transfected cells, neither in homomeric nor in heteromeric GlyR configurations. The EC_50_ values were increased for all GlyRα1^Q177^ variants in comparison to the wild type. The largest decrease in glycine potency was observed for the variant GlyRα1^Q177R^. Potencies of the partial agonists β-alanine and taurine were also reduced. Our data are further supported by homology modeling. The GlyRα1^Q177R^ variant does not form hydrogen bonds with the surrounding network of residue Q177 similar to the substitution with a basic lysine present in the mouse mutant *shaky*. Among all investigated Q177 mutants, the neutral exchange of glutamine to asparagine as well as the introduction of the closely related amino acid glutamic acid preserve the hydrogen bond network. Introduction of amino acids with small side chains or larger volume resulted in a loss of their hydrogen bonds to neighboring residues. The β8–β9 loop is thus an important structural and functional determinant of the inhibitory GlyR.

## Introduction

Glycine receptors (GlyRs) are predominantly expressed in the adult brain stem and spinal cord, where they represent the major component in inhibitory neurotransmission. GlyRs localized in motoneuron membranes in the spinal cord get activated upon glycine release from neighboring inhibitory interneurons. The chloride ion influx leads to hyperpolarization of motoneurons, balancing excitation of the muscle contraction (Rajendra et al., 1997). Disturbances in glycinergic inhibition are associated with rare disorders such as startle disease (OMIM 149400, hyperekplexia, stiff baby syndrome), pain (Harvey et al., 2004), autism spectrum disorders (Harvey and Yee, 2013; Pilorge et al., 2015), and panic disorders (Deckert et al., 2017). Human startle disease is caused by mutations in the *GLRA1, GLRB*, and *SLC6A5* genes encoding GlyR subunits α1 and β and the glycine transporter 2 (GlyT2).

Glycine receptors are described as homo- and heteropentameric ligand-gated ion channels of the superfamily of Cys-loop receptors (Lynch, 2004). For heteromeric GlyR complexes, five adjacent subunits are arranged around an ion channel pore composed of two α and three β subunits or three α and two β subunits (Grudzinska et al., 2005; Durisic et al., 2012; Yang et al., 2012). Each GlyR subunit consists of four TM helices, which are connected via intra- or extracellular loop structures. TM2 helices of all five subunits form the inner wall of the ion channel pore. The GlyR ECD is organized into an immunoglobulin-like structure and comprised of a short α-helix and 10 β-strands connected by loop structures (Du et al., 2015; Huang et al., 2015; Moraga-Cid et al., 2015). All Cys-loop receptors share the location of the agonist and antagonist-binding sites in the ECD formed by loops A, B, C from one subunit and loops D, E, F, G from an adjacent subunit (F loop in the following referred to as β8–β9 loop) (Brams et al., 2011). Ligands of the GlyR, starting with the highest affinity, are glycine, β-alanine, and taurine (Lynch, 2004). Antagonist of the GlyR is the alkaloid strychnine (C_21_H_22_N_2_O_2_) (Breitinger and Becker, 1998). Upon ligand binding, Cys-loop receptors get activated and undergo a variety of conformational changes and transition processes leading to tilts and slight turns of the TM helices and thus opening of the ion pore (Althoff et al., 2014).

Several *in vitro* studies revealed distinct contributions of ECD loop structures for receptor function. It was shown that phenylalanine 159 localized in the loop B contributes to a cation–π interaction with the incoming ligand, which is essential prior to channel opening (Pless et al., 2011). Moreover, loop C plays a role in transmitting the activation signal to the rest of the channel and exhibits a rearrangement upon ligand binding (Althoff et al., 2014). Mutations in the β2–β3 loop interfere with the maturation process of the protein and contribute to ligand specificity (Schaefer et al., 2015). Furthermore, loops β2–β3 and loop D determine ligand efficacy and play a role in ligand-induced desensitization or channel opening (Nys et al., 2013). The β8–β9 loop affects diazepam potentiation of the GABA_A_Rs (Padgett and Lummis, 2008). Loop β8–β9 has been suggested to play a major role in linking ligand binding to channel opening (Khatri and Weiss, 2010). Recently published structural models showed a coupling of movements within the ECDs, including the β8–β9 loop, proceeding to the TM helices resulting in their tilting and enabling ion channel opening and closing (Hassaine et al., 2014; Du et al., 2015; Huang et al., 2015).

We recently published the first *in vivo* model carrying a mutation within the β8–β9 loop (Schaefer et al., 2017). A single amino acid exchange GlyRα1^Q177K^ in the mouse mutant *shaky* resulted in premature death of homozygous animals. *In vivo*, synaptic GlyRs are decreased generating disturbed glycinergic signal transmission (Schaefer et al., 2017). At the structural level the hydrogen bond network around residue Q177 was disrupted.

Here, we investigated the role of side chain length, volume, and charge at amino acid position 177 in the β8–β9 loop of the GlyRα1. Our aim was to understand the importance of β8–β9 structural changes with respect to GlyR potency and gating. Our hypothesis is that neutral exchanges and amino acids that preserve the hydrogen network only marginally affect GlyR function. Therefore, we introduced the conservative amino acid exchange GlyRα1^Q177N^. The original mutation in the mouse mutant s*haky* was GlyRα1^Q177K^. We further generated the mutation of glutamine to arginine which is similar to the mutation in *shaky*, but the side chain volume is increased. The series was completed by introduction of small residues, e.g., glycine and alanine, or very large residues such as tryptophan. All residues preserving the hydrogen bond network around residue glutamine 177 had almost no effect on GlyR function. In contrast, residues that were unable to preserve the hydrogen bond network generated functional ion channels with impaired glycine potency. Thus, our data provide further evidence of the GlyR β8–β9 loop as a structurally but also functionally important element facilitating inhibitory neurotransmission in the adult organism.

## Materials and Methods

### Site-Directed Mutagenesis

PCR-mutagenesis was used to introduce the mutations (Q177A, Q177C, Q177D, Q177E, Q177G, Q177K, Q177N, Q177R, Q177W) at position 177 (numbering refers to mature protein). The murine GlyRα1 cDNA in the vector pRK7 was used as parental clone and refers to WT. Mutation-carrying amplimers were digested with Pst I and Bam HI and subcloned into the GlyRα1 WT sequence. All mutations were verified by sequencing (LGC Genomics, Berlin, Germany).

### Cell Lines

HEK293 human embryonic kidney cells CRL-1573, purchased from ATCC (Manassas, VA, United States) were grown in minimal essential medium (MEM) supplemented with 10% fetal calf serum, 200 mM GlutaMAX, 100 mM sodium pyruvate, and 50 U/ml penicillin/streptomycin (Thermo Fisher Scientific, Waltham, MA, United States) under standard growth conditions at 37°C and 5% CO_2_.

### Transfection

HEK293 cells were transiently transfected using a modified calcium phosphate precipitation method. A mixture of plasmid DNA, CaCl_2_, 0.1x TE buffer and 2x HBS (50 mM HEPES, 12 mM glucose, 10 mM KCl, 280 mM NaCl, 1.5 mM Na_2_HPO_4_) was applied onto the cells. A GlyRα1 to GlyRβ ratio of 1:2 was used for co-expression. For GlyRα1:GlyRβ:GFP co-expression, a ratio of 1:2:1 was transfected. The same amount of DNA was used for GlyRα1 WT and mutants. Media were exchanged after 6–24 h. Immunocytochemical stainings and electrophysiological experiments were always done 24 h after transfection, biotinylation experiments were performed 48 h after transfection.

### Cell Lysates

Whole cell lysates of transfected HEK293 cells transiently expressing the GlyRα1 variants and GlyRβ were acquired by using the CytoBuster Protein Extraction Reagent (Merck Millipore, Billerica, MA, United States) according to the manufacturer’s protocol. Forty micrograms of protein were diluted in 2x SDS sample buffer and heated for 5 min at 95°C before use in SDS-PAGE and Western blot.

### Biotinylation of Cell Surface Proteins

For biotinylation, transfected HEK293 cells transiently expressing the GlyRα1 variants or co-expressing GlyRβ were used. Medium was aspirated 48 h after transfection and cells were washed once with ice-cold PBS. To label surface proteins, cells were incubated with 1 mg/ml EZ-Link Sulfo-NHS-LC-Biotin (sulfosuccinimidyl-6-[biotin-amido]hexanoate) (Thermo Fisher Scientific, Waltham, MA, United States) for 30 min at 4°C. Cells were washed twice with ice-cold PBS, once with quenching buffer (192 mM glycine, 25 mM TRIS, in PBS pH 8.0) and incubated with quenching buffer for 10 min at 4°C. Cells were scraped into ice-cold PBS, centrifuged for 1 min at 1000 × *g*, 4°C, and lysed with 1% Triton X-100, 2 mM EDTA, 0.1 mM PMSF, and 10 mg/ml protease inhibitor (Roche, Basel, Switzerland) in TBS pH 8.0. The lysate was centrifuged again for 1 min at 17000 × *g*, 4°C. The supernatant (whole cell fraction, WC) was incubated with 50 μl of streptavidin agarose beads (Thermo Fisher Scientific, Waltham, MA, United States) using an overhead shaker for 2 h at 4°C. The supernatant (intracellular protein fraction) was removed and beads (surface protein fraction, SF) were washed three times with TBS buffer. The 60 μl 2x SDS sample buffer was added and the samples were heated 5 min at 95°C before use in SDS-PAGE and Western blot. Before gel loading, the protein amounts of the WC fraction were determined and 40 μg of protein loaded to each lane of the gel. For SF samples, the same volume (30 μl) was loaded.

### SDS-PAGE and Western Blotting

For protein separation, 11% PAM (polyacrylamide) gels were used. Gels were run at 150 V for 90 min. Proteins were transferred to nitrocellulose (GE Healthcare, Freiburg, Germany) using a wet blot transfer system (transfer buffer: 25 mM TRIS, 192 mM glycine, 10% ethanol) (Bio-Rad, Hercules, CA, United States). For GlyR protein transfer, 2 h at 200 mA were used. For larger proteins, e.g., cadherin, overnight blotting at 100 mA was performed. Membranes were blocked for 1 h with 5% BSA in TBS-T (TBS with 1% Tween 20). Primary antibodies were incubated overnight at 4°C. Proteins were detected with the pan-α antibody for GlyRs (mAb4a, Synaptic Systems, Göttingen, Germany, Cat. No. 146 011, 1:500) and pan-cadherin (Cell Signaling Technology, Danvers, MA, United States, Cat. No 4068, 1:1500) served as loading control. Signals were detected using the SuperSignal West (Thermo Fisher Scientific, Waltham, MA, United States).

### Immunocytochemical Staining

To stain GlyR surface receptors, GlyRα1 variants, and pDsRed-Monomer-Mem [Takara Bio (formerly Clontech), Mountain View, CA, United States] were co-transfected into HEK293 cells. All steps were performed at room temperature. After fixation for 20 min with 50 μl 4% paraformaldehyde, 4% sucrose solution, cells were washed three times with PBS and blocked for 30 min with 5% (v/v) goat serum in PBS. Afterward, cells were incubated for 1 h with the GlyRα1-specific primary antibody mAb2b (1:500 in blocking solution; epitope amino acids 1–10 of mature GlyRα1; Synaptic Systems, Göttingen, Germany). Cells were washed three times with PBS and incubated for 45 min with the secondary Alexa488-coupled goat-anti-mouse antibody (1:500 in blocking solution; Dianova, Hamburg, Germany). Then, cells were washed three times with PBS, incubated for 5 min with DAPI (1:5000 in PBS; Thermo Fisher Scientific, Waltham, MA, United States) and mounted on glass slides with Mowiol 4-88 (Carl Roth, Karlsruhe, Germany). Imaging was performed using an Olympus IX-81 inverted fluorescence microscope (Olympus, Tokyo, Japan).

### Confocal Microscopy, Image Acquisition, and Analysis

Images were acquired using an inverted Olympus IX81 microscope equipped with an Olympus FV1000 confocal laser scanning system, a FVD10 SPD spectral detector and diode lasers of 495 nm (Alexa488) and 550 nm (Cy3) (Olympus, Tokyo, Japan). All images shown were acquired with an Olympus UPLSAPO 60x (oil, numerical aperture: 1.35) objective. The images were further developed and organized by Adobe Photoshop (Adobe, San Jose, CA, United States) or ImageJ (1.51)/Fiji^1^.

### Electrophysiology

The patch clamp technique was used to measure current amplitudes (I) of transfected HEK293 cells. Currents were amplified using an EPC-9 amplifier and the software Patchmaster (HEKA, Lambrecht, Germany). Cells were patched 24 h after transfection in a whole-cell configuration mode. To measure glycine-evoked chloride currents, 1 mM or 100 μM glycine were applied. For EC_50_ measurements, glycine, β-alanine and taurine concentrations ranging from 1–3000 μM (glycine) or 3–10000 μM (β-alanine and taurine) were applied. All agonist and antagonists were applied using an Octaflow II system (ALA Scientific Instruments, Farmingdale, NY, United States). The extracellular buffer consisted of (in mM): 137 NaCl, 5.4 KCl, 1.8 CaCl_2_, 1 MgCl_2_, 5 HEPES, pH adjusted to 7.4 with NaOH. The intracellular buffer contained (in mM): 120 CsCl, 20 N(Et)_4_Cl, 1 CaCl_2_, 2 MgCl_2_, 11 EGTA, 10 HEPES, pH adjusted to 7.2 with CsOH. Recording pipettes with an open resistance of 4–6 MΩ were manufactured from borosilicate capillaries using a P97 horizontal puller (Sutter Instrument, Novato, CA, United States). Cells were held at -60 mV and fast capacitances were in range of 11–13 pF. All experiments were performed at room temperature.

### Statistical Analysis

Data analysis of Western blots: The image quantification was performed using ImageJ (1.51)/Fiji^2^. The data were analyzed using Student’s *t*-test (analysis of variance) and values below ^∗^*p* < 0.05 were considered significant, ^∗∗^*p* < 0.01, ^∗∗∗^*p* < 0.001. The values are displayed as means ± standard error of the mean (±SEM) or as otherwise noted. The graphs were generated using Origin 9.4 software (OriginLab, Northampton, MA, United States).

For the analysis of electrophysiological data, a non-linear algorithm (Origin, OriginLab, Northampton, MA, United States) was used to construct concentration-response curves from peak current amplitudes obtained with eight appropriately spaced concentrations in the range of 1–3000 μM glycine or 3–10000 μM β-alanine, and taurine. The following Hill equation was used: *I = I*_max_ *^∗^ c^nHill^/(c^nHill^ + EC*_50_*^nHill^)*. *I* refers to the current amplitude at the given agonist concentration *c, I*_max_ is the current amplitude at a saturating agonist, *EC*_50_ refers to the agonist concentration evoking half-maximal current responses and *n_Hill_* is the Hill coefficient.

Images were processed with ImageJ (1.51)/Fiji^2^ (Schindelin et al., 2012, 2015; Schneider et al., 2012) and Adobe Photoshop (Adobe, San Jose, CA, United States).

### Computational Methods

The effect of different mutations was predicted based on the recent structure of the human homopentameric GlyRα3 in complex with AM-3607 and glycine (Huang et al., 2017). Mutations were introduced *in silico* in the structure and the side chains were positioned in preferred rotamer conformations while minimizing steric overlaps as well as optimizing hydrogen-bonding capabilities using the software Coot (Emsley et al., 2010). Images were made using The PyMOL Molecular Graphics System, Version 1.8 Schrödinger, LLC.

For the alignment the sequences of human GlyRα1, murine GlyRα1/α2/α3/β, and GABA_A_ α1/γ2 were aligned with Clustal Omega using the default settings^3^ (Sievers et al., 2011).

## Results

Our current understanding of GlyR ion channel opening and closing suggests concerted movements within the ECD (loops β9–β10, β1–β2, and β6–β7) upon ligand-binding that are transmitted to elements of the ECD-TMD interface (β10-pre-M1, the M2-3 loop) (Du et al., 2015). The β8–β9 loop is part of the ligand-binding site by its localization underneath the ligand-binding pocket (Brejc et al., 2001; Hansen et al., 2005). Recently, we found in a novel startle disease mouse model that a β8–β9 loop alteration disrupts GlyR ligand-binding site stability and ion channel gating (Schaefer et al., 2017).

Here, we investigated the role of side chain volume and charge at position Q177 within the β8–β9 loop for GlyR expression, agonist potency and structural consequences within the hydrogen bond network of the GlyR ECD.

### GlyRα1 Amino Acid Substitutions at Position Glutamine 177 in the β8–β9 Loop

**FIGURE 1:**
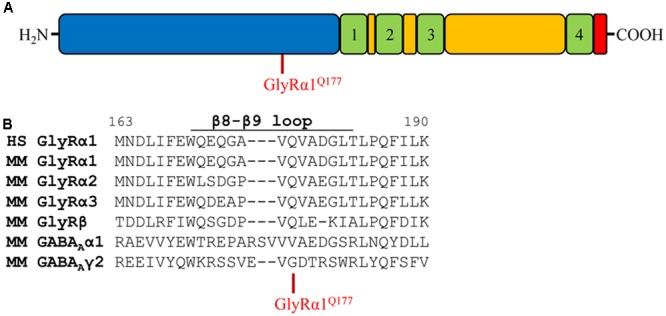
The glycine receptor β8–β9 loop. **(A)** Schematic view of the GlyR domain structure containing the N-terminus (blue), transmembrane domains 1–4 (green) connected by loops (yellow), and C-terminus (red). Residue GlyRα1^Q177^ highlighted in red. **(B)** Sequence alignment of the β8–β9 loop and adjacent residues of human (HS) GlyR α1, mouse (MM) GlyR α1, α2, α3, β and mouse GABA_A_ α1, γ2 subunits.

Glycine receptors share a large organized N-terminal domain (ECD) with other Cys-loop receptors. The ECD consists of an α-helix, followed by 10 β-strands connected by loop structures important for transmitting conformational changes upon ligand-binding. Residue 177 is localized in the β8–β9 loop and occupied by a glutamine in all murine GlyRα subunits, GlyRβ as well as human GlyRα1 (**Figures 1A,B**). The closely related GABA_A_ receptor subunits α1 and γ2 carry a valine (α1) or a glycine (γ2) at the corresponding residue in the β8–β9 loop (**Figure 1B**). Mutations within GlyR subunits are associated with human hyperekplexia (Startle disease). The amino acid exchange of Q177 into a lysine resulted in severe startle disease in the mouse mutant *shaky* (Schaefer et al., 2017).

Apart from the GlyRα1^Q177K^ mutation in the mouse mutant *shaky*, the following amino acids were introduced at position 177: arginine (GlyRα1^Q177R^) carrying a positively charged side chain similar to lysine in the *shaky* mutant; asparagine (GlyRα1^Q177N^) with a hydrophilic side chain and similar to the original glutamine; aspartate and glutamate (GlyRα1^Q177D^, GlyRα1^Q177E^) both negatively charged; glycine (GlyRα1^Q177G^) a neutral and small amino acid (present in GABA receptor γ2); alanine (GlyRα1^Q177A^) with a small hydrophobic side chain; cysteine (GlyRα1^Q177C^) with a thiol side chain; and tryptophan (GlyRα1^Q177W^) carrying a sterically demanding side chain.

### β8–β9 Loop Alterations with Positive Charged Side Chains at Position Q177 Reduce Surface Expression *In Vitro*

Glycine receptor mutants associated with startle disease affect either GlyR expression or GlyR function. Mutation of GlyRα1^Q177K^ resulted in reduced α1 expression *in vitro* [33 ± 5% of wild-type α1β (Schaefer et al., 2017)].

**FIGURE 2:**
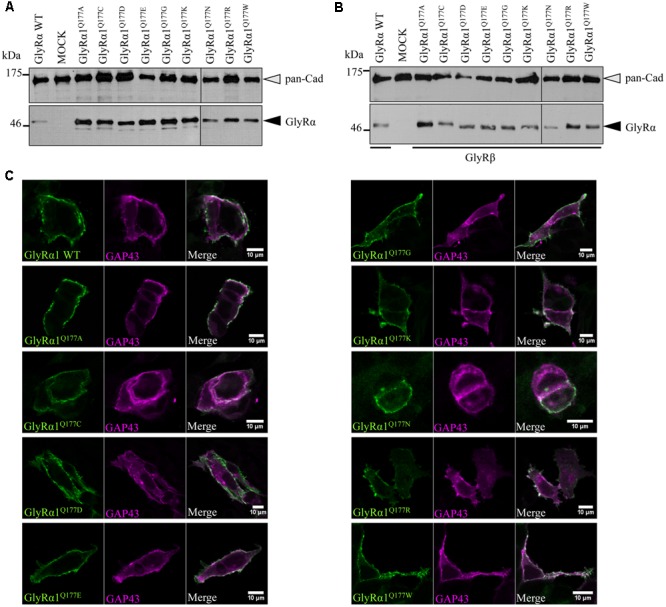
GlyRα1 Q177 variants are expressed. **(A,B)** Crude whole cell lysates of HEK293 cells transiently expressing GlyRα1 WT or GlyRα1^Q177^ variants without GlyRβ **(A)** or with GlyRβ **(B)** co-expression (black arrowhead, mAb4a antibody, 48 kDa). Cells transfected with only GFP served as negative control (MOCK) and pan-Cadherin (gray arrowhead, pan-Cadherin antibody, 135 kDa) was used as loading control. **(C)** HEK293 cells were co-transfected with GlyRα1 WT or GlyRα1^Q177^ variants and a plasma membrane marker (GAP43 fused to dsRed, false color magenta). GlyRα1 WT and GlyRα1^Q177^ variants were detected with the GlyR α1-specific mAb2b antibody (green) using cell staining to label surface receptors only. The overlay between GAP-43 and GlyRα1 WT or GlyRα1^Q177^ variants is shown in white (right column). The white bar refers to 10 μM.

Analysis of crude protein lysates (**Figures 2A,B**) showed protein expression of all GlyRα1^Q177^ variants with no overall differences whether co-expressed with GlyRβ or when expressed alone. In addition, cell stainings for surface expression also revealed no obvious differences between GlyRα1 WT and GlyRα1^Q177^ variants (**Figure 2C**). GAP-43 expressed as a fusion protein with dsRed encoded on a co-transfected plasmid was used as membrane marker and for control of transfection efficiency. All GlyRα1^Q177^ variants exhibited colocalization with GAP-43 and thus cellular surface expression.

**FIGURE 3:**
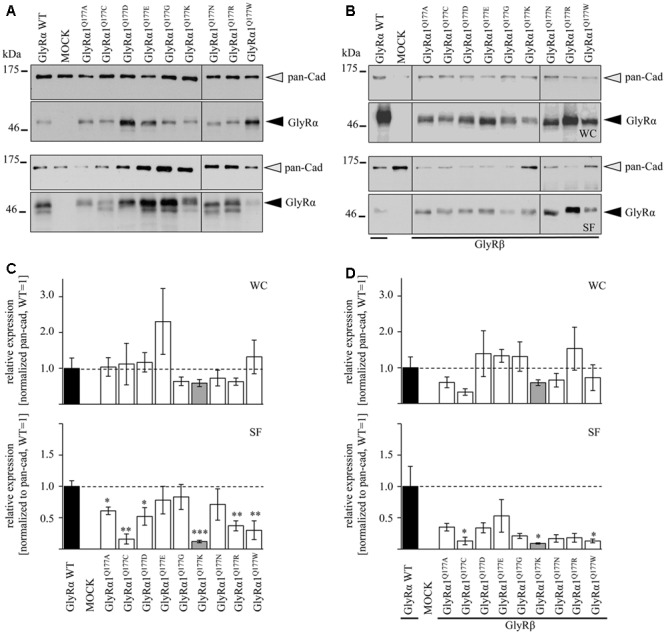
The surface expression is influenced by mutations at position GlyRα1^Q177^. **(A,B)** Biotinylation assays were performed to quantify the expression of GlyRα1^Q177^ variants and distinguish between whole cell (WC) and surface (SF) proteins level. Samples were analyzed by Western blotting using the GlyR pan-α antibody mAb4a (black arrowhead, 48 kDa). Pan-cadherin served as loading control (gray arrowhead, 135 kDa). Cells transfected with only GFP (MOCK) were used as negative control. Three to four independent experiments were performed. Note, an enhanced degradation for variants GlyRα1^Q177D^, GlyRα1^Q177R^, and GlyRα1^Q177W^. Vertical lines indicate that protein bands were taken from different gels of the same experiment. **(C,D)** WC and SF protein levels of independent biotinylation assays without GlyRβ **(C)** or with GlyRβ **(D)** co-expression were normalized to the loading control pan-cadherin of the same sample and further correlated to GlyRα1 WT expression which was set to 1 (see dotted line in **(C)** and **(D)**; equals 100%, **Table 1**). Error bars refer to the standard error of the mean (SEM). Level of significance refer to ^∗^*p* < 0.05, ^∗∗^*p* < 0.01, ^∗∗∗^*p* < 0.001.

Protein quantification from Western blots after pull-down of biotinylated surface proteins by streptavidin-beads concomitantly demonstrated no significant differences in whole cell (WC) expression for GlyRα1^Q177^ variants (**Figures 3A** upper panel, **C** and **Table 1**) or GlyRα1^Q177^ variants coexpressed with GlyRβ subunit (**Figures 3B** upper panel, **D** and **Table 1**). The biotinylation method also allows a direct comparison between WC and surface protein fraction (SF) (**Figures 3A,B** lower panel and **Table 1**). Note, in the surface fractions of variants GlyRα1 enhanced degradation is visible but also for WT receptors. Only the upper band was used for calculation of the surface protein amount. Degradation might result from maturation deficits, showing that GlyRα1^Q177^ variants seem to get stuck on their way to the cell surface, most probably in the ER compartment as shown previously for other recessive GlyRα1 variants (Schaefer et al., 2015). However, significant differences of SF protein level were observed for some GlyRα1^Q177^ variants without or with co-expression with GlyRβ (**Figures 3C,D**). The GlyR expression of the Q177 variants was normalized to expression of pan-cadherin in the same sample serving as a membrane marker control protein. The resulting relative expression of GlyRα1WT was set to 1 (corresponding to 100%) and the relative expression of the GlyRα1 variants calculated accordingly (**Figures 3C,D** lower panels and **Table 1**).

**Table 1:** Protein expression profile of GlyRα1^Q177^ variants.

Construct	Number (*n*)	Whole cell rel. expression normalized to pan-cad	Significance ^∗^*p* < 0.05, ^∗∗^*p* < 0.01, ^∗∗∗^*p* < 0.001	Whole cell rel. expression normalized to pan-cad [%]	Number (*n*)	Surface rel. expression normalized to pan-cad	Significance ^∗^*p* < 0.05, ^∗∗^*p* < 0.01, ^∗∗∗^*p* < 0.001	Surface rel. expression normalized to pan-cad [%]
**Single expression**	**mAb4a signal (N-domain)**							

GlyRα1 WT	4	0.78 ± 0.23		100 ± 30	4	3.02 ± 0.28		100 ± 9

GlyRα1^Q177A^	4	0.81 ± 0.2	n.s.	102 ± 25	3	1.85 ± 0.19	^∗^	61 ± 6
GlyRα1^Q177C^	4	0.87 ± 0.45	n.s.	112 ± 58	3	0.47 ± 0.24	^∗∗^	16 ± 8
GlyRα1^Q177D^	4	0.91 ± 0.21	n.s.	116 ± 27	4	1.57 ± 0.43	^∗^	52 ± 14
GlyRα1^Q177E^	4	1.8 ± 0.72	n.s.	230 ± 92	3	2.35 ± 0.65	n.s.	78 ± 22
GlyRα1^Q177G^	4	0.5 ± 0.09	n.s.	64 ± 12	3	2.51 ± 0.61	n.s.	84 ± 20
**GlyR**α**1^Q177K^**	4	0.46 ± 0.08	n.s.	59 ± 10	3	0.36 ± 0.06	^∗∗∗^	12 ± 2
GlyRα1^Q177N^	4	0.57 ± 0.17	n.s.	73 ± 22	3	2.13 ± 0.76	n.s.	71 ± 25
GlyRα1^Q177R^	4	0.49 ± 0.08	n.s.	63 ± 10	4	1.12 ± 0.23	^∗∗^	37 ± 8
GlyRα1^Q177W^	4	1.03 ± 0.37	n.s.	132 ± 46	3	0.91 ± 0.44	^∗∗^	30 ± 15

**Co-expression with GlyRβ WT**	**mAb4a signal (N-domain)**							

GlyRα1 WT	5	0.97 ± 0.29		100 ± 30	3	3.36 ± 1.09		100 ± 33

GlyRα1^Q177A^	4	0.57 ± 0.15	n.s.	59 ± 15	3	1.17 ± 0.19	n.s.	35 ± 6
GlyRα1^Q177C^	4	0.31 ± 0.09	n.s.	31 ± 9	3	0.44 ± 0.2	^∗^	13 ± 6
GlyRα1^Q177D^	4	1.35 ± 0.62	n.s.	139 ± 64	3	1.13 ± 0.27	n.s.	33 ± 8
GlyRα1^Q177E^	4	1.29 ± 0.17	n.s.	133 ± 17	3	1.79 ± 0.88	n.s.	54 ± 27
GlyRα1^Q177G^	4	1.27 ± 0.4	n.s.	130 ± 41	3	0.71 ± 0.13	n.s.	22 ± 4
**GlyR**α**1^Q177K^**	4	0.56 ± 0.08	n.s.	58 ± 8	3	0.30 ± 0.03	^∗^	9 ± 1
GlyRα1^Q177N^	4	0.63 ± 0.18	n.s.	65 ± 18	3	0.56 ± 0.21	n.s.	17 ± 6
GlyRα1^Q177R^	4	1.48 ± 0.58	n.s.	152 ± 60	3	0.59 ± 0.23	n.s.	18 ± 7
GlyRα1^Q177W^	4	0.7 ± 0.35	n.s.	72 ± 36	3	0.44 ± 0.11	^∗^	13 ± 3

*To calculate the relative expression in percent (%), the WT expression was set to 100%. All other estimated relative expression values were calculated accordingly. n.s., non-significant.*

In single expression studies, the SF protein level for GlyRα1^Q177A^ (61 ± 6%), GlyRα1^Q177C^ (16 ± 8%), GlyRα1^Q177D^ (52 ± 14%), GlyRα1^Q177K^ (12 ± 2%), GlyRα1^Q177R^ (37 ± 8%), and GlyRα1^Q177W^ (30 ± 15%) were significantly reduced compared to α1WT (**Figure 3C** and **Table 1**). Variants GlyRα1^Q177E^, GlyRα1^Q177G^, GlyRα1^Q177N^ exhibited surface expression indistinguishable from GlyRα1WT (**Table 1**).

In co-expressions with GlyRβ, the surface expression levels were lower compared to single expressions. The GlyR complex consists of two α and three β subunits (Grudzinska et al., 2005) which coassemble within the endoplasmic reticulum. We coexpressed α1 and β in a 1:2 ratio. The low amount of the β subunit during transfection was probably the reason for the lower expression of GlyRα1 variants at the cell surface in α1β co-expressions (**Figures 3C,D**).

Surface fraction protein level for co-expression with GlyRβ demonstrated significant changes: GlyRα1^Q177C^ (13 ± 6%), GlyRα1^Q177K^ (9 ± 1%), and GlyRα1^Q177W^ (13 ± 3%) (**Figure 3D** and **Table 1**). Interestingly, the conservative exchange of an asparagine instead of the GlyRα1 WT glutamine had no impact on whole cell and surface expression. The expression of variant GlyRα1^Q177E^ was also similar to GlyRα1 WT and also did not lead to significant differences in GlyR expression level at the cell surface.

### β8–β9 Loop Variants Reduce Potency of the Agonist Glycine

Changes in the integrity of the β8–β9 loop have been determined to reduce glycine potency and modify the function as a gating element of this receptor class (Schaefer et al., 2017).

**FIGURE 4:**
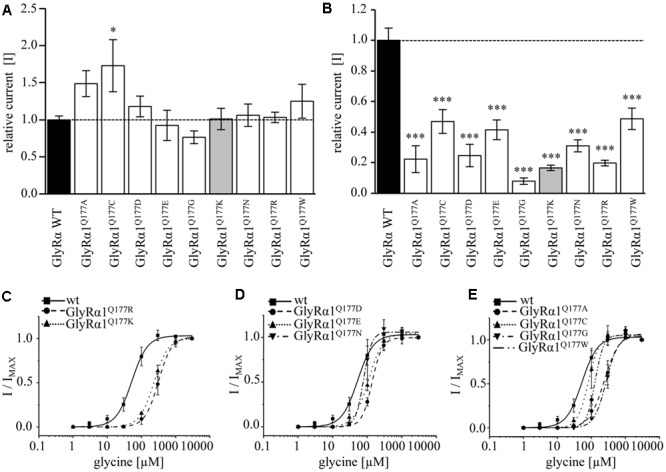
β8–β9 GlyRα1^Q177^ variants lead to changes in ion channel characteristics. All electrophysiological recordings were performed from transfected HEK293 cells expressing αβ heteromeric receptors with GlyRα1 mutants or WT and GlyRβ WT. Functional parameters from whole-cell recordings of transfected HEK293 cells with GlyRα1β WT or GlyRα1^Q177^β variants, mean values of current amplitudes at 1 mM **(A)** and 100 μM **(B)** of glycine (*n* = 5–8 cells) are shown. The mean of GlyRα1β WT was set to 1 (marked by dotted line). Level of significance ^∗^*p* < 0.05, ^∗∗∗^*p* < 0.001. **(C–E)** Ligand binding potencies (EC_50_) determined by whole-cell current measurements of transfected cells expressing GlyRα1β WT or GlyRα1^Q177^β variants with eight different glycine concentrations (1–3000 μM, *n* = 3–5 for each receptor configuration).

Glycine receptor physiology of the mutant receptors coexpressed with the GlyRβ subunit was determined by electrophysiological measurements using whole-cell recordings following transient expression in HEK293 cells. As a preliminary screen, absolute currents values (I) were determined at saturating concentrations of glycine (1 mM) and at a glycine concentration around the EC_50_ value of the GlyRα1β WT (100 μM). No significant differences in *I*_max_ values for all GlyRα1^Q177^β variants in comparison to GlyRα1β WT were observed (**Figure 4A**) but significant reductions in glycine-induced currents at 100 μM glycine (**Figure 4B** and **Table 2**).

**Table 2:** Electrophysiological properties of GlyRα1^Q177^ variants.

α1 construct co-expressed with GlyRβ	Number of cells (n)	Mean*I*_gly 100 μM_ [nA] ± SEM	Number of cells for EC_50_ glycine	EC_50_ glycine [μM ± SEM]	*n*_H_ glycine	Number of cells for EC_50_ β-alanine	EC_50_ β-alanine [μM ± SEM]	*n*Hβ-alanine	Number of cells for EC_50_ taurine	EC_50_ taurine μM ± SEM]	*n*H taurine
GlyRα1 WT	30	3.8 ± 0.3	5	58 ± 4	1.9	3	157 ± 7	2.6	3	593 ± 18	2.3

GlyRα1^Q177A^	6	0.8 ± 0.3***	4	208 ± 22	2.0		ND			ND	
GlyRα1^Q177C^	8	1.8 ± 0.3***	3	88 ± 3	2.1		ND			ND	
GlyRα1^Q177D^	6	0.9 ± 0.3***	5	137 ± 4	3.4		ND			ND	
GlyRα1^Q177E^	5	1.6 ± 0.2***	4	109 ± 7	2.6		ND			ND	
GlyRα1^Q177G^	5	0.3 ± 0.08***	3	262 ± 13	2.5		ND			ND	
**GlyR**α**1^Q177K^**	**5**	**0.6 ± 0.07^∗∗∗^**	**3**	**312 ± 7**	**2.2**		**ND**			**ND**	
GlyRα1^Q177N^	5	1.2 ± 0.2***	4	96 ± 1	3.0	3	203 ± 17	1.7	3	1254 ± 72	1.6
GlyRα1^Q177R^	5	0.8 ± 0.07***	4	231 ± 4	2.0	3	359 ± 19	1.9	3	2982 ± 183	1.5
GlyRα1^Q177W^	5	1.8 ± 0.3***	3	130 ± 12	3.7		ND			ND	

*Significance values = *p*; ^∗∗∗^*p* < 0.001, *n*, number of cells recorded, *n*_*H*_ hill coefficient, SEM, standard error of the mean; ND, not determined.*

These measurements were followed by a determination of a dose–response curve using eight different glycine concentrations (1, 3, 10, 30, 100, 300, 1000, and 3000 μM) to determines changes in glycine potency for GlyRα1^Q177^β variants. An EC_50_ of 58 ± 4 μM for GlyRα1β WT was estimated in transfected HEK293 cells (ratio of 1:2 α1:β, **Figure 4C**). The glycine EC_50_ values for GlyRα1^Q177^β variants differed in a range between a two-fold (GlyRα1^Q177N^β or GlyRα1^Q177C^β) up to a six-fold increase (GlyRα1^Q177K^β) (**Figures 4C–E** and **Table 2**). Variants were grouped according to structural characteristics. Group one is comprised of GlyRα1^Q177K^β and GlyRα1^Q177R^β, both positive amino acids which showed a large increase in EC_50_ when compared to GlyRα1β WT (GlyRα1^Q177K^β 312 ± 7 μM, a 5.3-fold increase and GlyRα1^Q177R^β 231 ± 4 μM, a 4-fold increase) (**Figure 4C**). The second group consists of the aspartate mutant (negatively charged), the glutamic acid mutant (negatively charged) and the conservative asparagine (polar). The observed increase in EC_50_, was minor for GlyRα1^Q177D^β 137 ± 4 μM, a 2.3-fold increase; GlyRα1^Q177E^β 109 ± 7 μM, a 1.8-fold increase; GlyRα1^Q177N^β 96 ± 1 μM, a 1.6-fold increase) (**Figure 4D**). The third group includes GlyRα1^Q177A^β, GlyRα1^Q177C^β, GlyRα1^Q177G^β, GlyRα1^Q177W^β which all showed increased EC_50_ values: GlyRα1^Q177A^β 208 ± 22 μM, a 3.6-fold increase; GlyRα1^Q177G^β 262 ± 13 μM, a 4.5-fold increase and GlyRα1^Q177W^β 130 ± 12 μM, a 2.2-fold increase) (**Figure 4E**). Interestingly, the substituted cysteine with an overall size close to glutamine but rather different to the small residues glycine and alanine or the bulky tryptophan exhibited only a slight shift in glycine potency (88 ± 3 μM, a 1.5-fold increase).

To further discriminate between agonist potency and gating defects, we used the GlyRα1 asparagine mutant (GlyRα1^Q177N^), which is closely related to the original glutamine and the arginine mutant (GlyRα1^Q177R^) similar to the lysine mutants in the mouse mutant *shaky.* These mutants were analyzed upon application of the partial GlyR agonists β-alanine and taurine.

**FIGURE 5:**
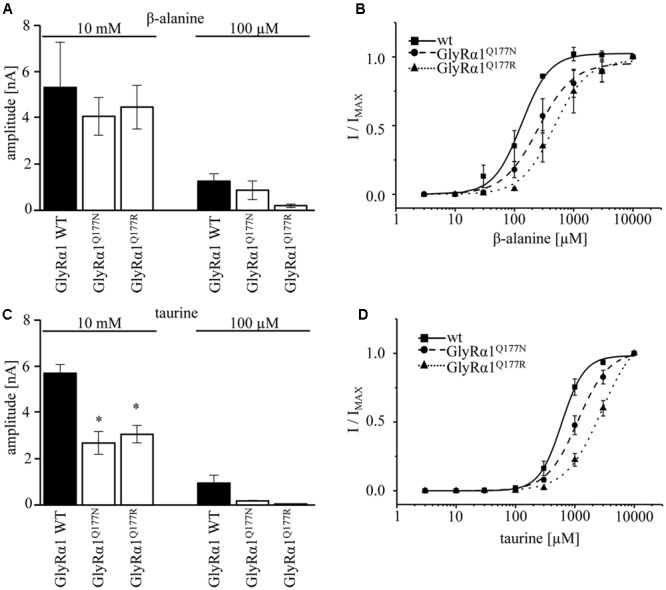
GlyRα1^Q177^ variants result in changes in potency and efficacy of the partial GlyR agonists β-alanine and taurine. GlyRα1^Q177^ variants were coexpressed with GlyRβ WT. **(A)** Comparison of the absolute current amplitudes (*I*) between GlyRα1 WT, GlyRα1^Q177N^, GlyRα1^Q177R^ at a saturating β-alanine concentration of 10 mM and at 100 μM. **(B)** Dose–response curves for GlyRα1 WT and the GlyRα1 mutants using β-alanine at different concentrations (3–10000 μM). **(C)** Determination of taurine efficacy at saturating concentration of taurine 10 mM and at 100 μM. Absolute current amplitudes (*I*) are shown; level of significance ^∗^*p* < 0.05. **(D)** Dose–response curves of taurine (3–10000 μM) for GlyRα1 WT, GlyRα1^Q177N^, and GlyRα1^Q177R^.

A concentration of 100 μM for both partial agonists generated non-significant changes in agonist-induced inward currents with 1.3 ± 0.3 nA for GlyRα1β WT, 0.87 ± 0.4 nA for GlyRα1^Q177N^β, and 0.2 ± 0.07 nA for GlyRα1^Q177R^β at 100 μM β-alanine and 0.13 ± 0.06 nA for GlyRα1β WT, 0.02 ± 0.001 nA for GlyRα1^Q177N^β, and 0 ± 0 nA for GlyRα1^Q177R^β at 100 μM taurine (**Figures 5A,C**).

The application of 10 mM β-alanine did not result in significant differences between GlyRα1β WT (5.3 ± 2 nA, *n* = 3) and GlyRα1^Q177N^β (4.1 ± 0.8 nA, *n* = 3) or GlyRα1^Q177R^β 4.5 ± 0.9 nA, *n* = 3) (**Figure 5A**). In contrast, taurine application at 10 mM significantly reduced GlyR efficacy with GlyRα1β WT 5.7 ± 0.4 nA, *n* = 3; GlyRα1^Q177N^β 2.7 ± 0.5 nA, *n* = 3; and GlyRα1^Q177R^β 3.1 ± 0.4 nA, *n* = 3 (**Figure 5C**). Although, the GlyRα1β WT reached saturation at 10 mM taurine, it is obvious that the mutants probably not completely reached saturation.

Hence, we further examined β-alanine potency in comparison to GlyRα1 WT, which exhibited a slight increase of the EC_50_ for β-alanine (GlyRα1β WT 157 ± 7 μM; GlyRα1^Q177N^β 203 ± 17 μM a 1.3-fold increase; GlyRα1^Q177R^ 359 ± 19 μM, a 2.3-fold increase) (**Figure 5B** and **Table 2**). For taurine potency, the EC_50_ for the arginine and asparagine GlyRα1 variants showed the following results in comparison to GlyRα1β WT (GlyRα1β WT 593 ± 18 μM; GlyRα1^Q177N^β 1254 ± 72 μM, a 2.1-fold increase; and GlyRα1^Q177R^β 2982 ± 183 μM, a 5-fold increase) (**Figure 5D** and **Table 2**).

In conclusion, the physiological data point to a mixed phenotype affecting agonist/partial agonist potency and most probably partial agonist efficacy at least for taurine.

### Structural Modeling Revealed Changes in the Hydrogen Bonding Pattern of β8–β9 Mutants

**FIGURE 6:**
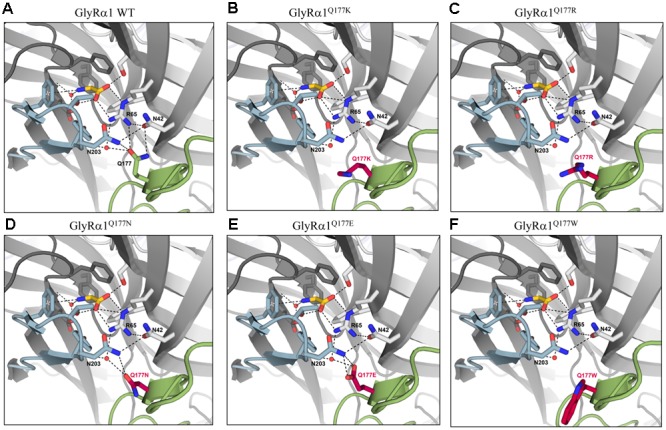
Q177 is localized in a hydrogen bond network important for GlyR function. Q177 is part of a hydrogen-bond network with R65, a critical residue in glycine binding. View into the ligand binding site in the crystal structure of GlyRα3 in complex with AM-3607 and glycine ((Huang et al., 2017) PDB code 5TIN). The principal and complementary subunits are colored gray, respectively. The agonist glycine is shown in yellow, residues that form the ligand binding pocket are marked in blue, Q177 in green or modeled variants **(A)** GlyRα1 WT, **(B)** GlyRα1^Q177K^, **(C)** GlyRα1^Q177R^, **(D)** GlyRα1^Q177N^, **(E)** GlyRα1^Q177E^, and **(F)** GlyRα1^Q177W^ at this position in red. Loops C and β8–β9 are marked in light blue (loop C) and light green (β8–β9). Residues that are engaged in the hydrogen bond network are shown as sticks, relevant water molecules are represented as spheres. Hydrogen bonds (to max. distance of 3.3 Å) are indicated as black dashed lines.

Earlier studies reported the importance of the hydrogen bond network within the GlyR ECD or the closely related GABA_A_ receptor for stabilization of the structure (Padgett and Lummis, 2008; Yu et al., 2014). The mutation GlyRα1^Q177K^ disrupts the hydrogen bond network around residue Q177 (**Figures 6A,B**). Similarly, the introduction of an arginine at position 177 resulted in lack of hydrogen bonds to neighboring residues such as N42, R65, and N203 (**Figure 6C**). Interestingly residues that marginally affect GlyR function, such as GlyRα1^Q1777E^ and GlyRα1^Q177N^ were still able to form a hydrogen bond, arguing for minor structural changes to the GlyR structure (**Figures 6D,E**). Not in line with a direct correlation between a lack of the hydrogen bond network and large changes in glycine potency are the data on GlyRα1^Q177C^. This residue is predicted to lack the hydrogen bonds to R65 and N203 (data not shown), but exhibited only small changes in glycine potency. From its size, cysteine is similar to asparagine. One might therefore argue that size and side chain property underlie the observed effects on glycine potency.

The large side chain volume of GlyRα1^Q177W^ is expected to completely disrupt the hydrogen bond network (**Figure 6F**). Similarly, this amino acid exchange resulted in an increased EC_50_ for the agonist glycine. The correct side chain volume of glutamine at position 177 seems to be critical since tryptophan, the bulkiest side chain, and glycine, the smallest residue, behaved similarly. Glycine most probably is too small and lacks side chain atoms which can engage in the formation of hydrogen bonds, while in the larger residues, the bulkier side chain is no longer able to interact with N42, R65, and N203 (**Figure 6F**).

## Discussion

Signal transduction from ligand-binding into channel opening in Cys-loop receptors involves concerted conformational changes of defined structures of the ECD at both the principle (+) and the complementary (-) site of the intersubunit interface (Hilf and Dutzler, 2008; Hibbs and Gouaux, 2011; Du et al., 2015; Morales-Perez et al., 2016). The involvement of the β8–β9 loop in these conformational rearrangements was first described in the cryo-EM structure of the GlyRα1 subunit analyzing transitions between the closed receptor configuration and the open states (Du et al., 2015).

Previous studies on Cys-loop receptors determined a hydrogen bond network of the β8–β9 loop with residues close to the ligand binding site as important for transitions between different receptor states (Padgett and Lummis, 2008; Nys et al., 2013; Yu et al., 2014). Disruptions in structurally important GlyR elements may underlie disease pathology of startle disease (Bode and Lynch, 2014). Several mutations distributed all over the GlyR structure have been correlated to disease mechanisms (Chung et al., 2010).

Within the β8–β9 loop one human mutation was so far identified GlyRα1^W170S^ (Al-Futaisi et al., 2012; Zhou et al., 2013). An RNA-editing variant P185L of the GlyRα3 β8–β9 loop detected in patients with mesial temporal lobe epilepsy (TLE) has been described to increase glycine potency *in vitro* (Meier et al., 2005; Eichler et al., 2009). The murine GlyRα1 startle disease mutant *shaky* (Q177K) resulted in lethality of homozygous animals due to a complex functional pattern including reduced synaptic expression, decreased agonist potency and accelerated ion channel closure (Schaefer et al., 2017). Moreover, glutamine 177 is part of the hydrogen bond network important for ion channel function. Here, we investigated the side chain volume and charge of Q177 with regard to expression and ion channel functionality.

In startle disease, depending on the type of mutation either ion channel function (dominant trait) or expression and transport (recessive mutation) is disabled (Chung et al., 2010; Bode and Lynch, 2014). Although GlyRα1^Q177K^ leads to decreased expression *in vitro*, there is still enough protein expressed to generate functional ion channels (Schaefer et al., 2017).

For all GlyRα1^Q177^ variants, the overall expression levels were indistinguishable from α1WT. Using protein quantification analyses combined with discrimination between whole cell and surface protein, differences between GlyRα1 WT and the mutants were identified. In contrast to no significant differences in the whole cell protein amount of all GlyRα1^Q177^ variants, surface expression levels differed between WT and mutants.

Similar to the *shaky* variant, GlyRα1^Q177K^, the mutant GlyRα1^Q177R^ showed a decreased expression when expressed alone or in co-expression with GlyRβ. The conservative exchange of glutamine 177 to asparagine, however, did not result in a different expression pattern in comparison to GlyRα1 WT. Interestingly, the introduction of a negative charge, such as in GlyRα1^Q177E^ which is a structurally similar amino acid residue to the original glutamine resulted in a similar expression pattern like GlyRα1 WT. The introduction of neutral amino acid residues either small (glycine, alanine, and cysteine) or bulky (tryptophan) resulted in reduced surface receptor levels, independent of the presence of the β-subunit.

A reduction of cell surface receptors argues for differences of maximal currents upon application of saturating glycine concentration. Earlier reports on recessive hyperekplexia mutants demonstrated that a reduction of cellular membrane expression revealed smaller *I*_max_ values in electrophysiological recordings (Villmann et al., 2009; Chung et al., 2010). The *shaky* mutant GlyRα1^Q177K^, however, did not result in changes at glycine-induced currents at saturating glycine concentrations although the surface expression was reduced *in vitro* (Schaefer et al., 2017). Here, at a saturating concentration of glycine, all GlyRα1^Q177^ variants exhibited maximal current amplitudes almost indistinguishable from WT. The estimated reduction of cell surface expression for the GlyRα1^Q177^ mutants is thus not sufficient to change the maximal current amplitudes in the HEK293 cell overexpression system.

Due to decreased glycinergic currents at lower glycine concentrations observed for all GlyRα1^Q177^ mutants, a reduction of glycine potency was suggested. Glycine potency was differently affected in GlyRα1 variants. A lower glycine potency was exhibited by GlyRα1^Q177R^. The determined four-fold decrease of glycine potency was similar to the *shaky* mutation GlyRα1^Q177K^ (Schaefer et al., 2017). Small changes were observed for the conservative exchange of glutamine to asparagine (1.6-fold) as well as to the charged glutamate (1.8-fold). The presence of the smallest amino acids glycine and alanine at position 177 revealed higher EC_50_ values for the agonist glycine which were similar to the EC_50_ of the very bulky residue tryptophan.

The β8–β9 loop is not directly involved in ligand binding but in structural transitions between agonist bound receptor and the open state of the ion channel (Althoff et al., 2014; Du et al., 2015). No changes in agonist/antagonist affinities have been observed in the *shaky* mouse carrying the GlyRα1^Q177K^ mutation. Glycine and strychnine bound with the same efficiency to the mutated receptor compared to GlyRα1 WT (Schaefer et al., 2017). The observed glycine potency has been determined to correlate with changes in the hydrogen bond network around residue Q177.

In the variants GlyRα1^Q177K^ or GlyRα1^Q177R^ the hydrogen bond with residue R65 is disrupted. In the modeled GlyRα1 WT, the oxygen of the amide at the side chain of glutamine acts as the hydrogen bond acceptor. The side chains of lysine and arginine lack this oxygen, which in turn hinder GlyRα1^Q177K^ or GlyRα1^Q177R^ to form a hydrogen bond with R65. Furthermore, the side chains of lysine and arginine are longer compared to glutamine, arguing for steric effects that might be transferred to conformational changes to the ligand-binding site and thus explain differences in glycine potency.

Asparagine is almost identical in structure to the original glutamine (Q177) in the β8–β9 loop with the side chain missing one methylene unit, so one would expect results similar to the GlyRα1 WT. The shorter side-chain of asparagine is predicted to result in a weaker hydrogen bond between GlyRα1^Q177N^ and R65, as shown in the structural modeling, resulting in a different conformation of the ligand-binding site which probably destabilizes the glycine binding pocket.

While glutamine features an amide, in contrast, glutamic acid carries a carboxyl group. Aspartic acid also features a carboxyl group instead of an amide and misses a methylene unit compared to glutamine. A hydrogen bond between R65 and GlyRα1^Q177D^ or GlyRα1^Q177E^ should be possible with an oxygen of the carboxyl group acting as acceptor. However, the negative side chain charge may affect the strength of the hydrogen bond and also the interaction with other nearby structural elements, e.g., N42. Modeling of the putative position of the glutamate side chain showed an orientation toward the amide group of N203, to which it can form tight hydrogen bonds. In contrast, this repositioning moves the side chain further away from R65 and thereby a second hydrogen bond is lost.

Although, the amino acids glycine and tryptophan differ the most in side chain volume, the determined glycine potency was similarly affected. Glycine has no side chain. Hence, no hydrogen bond between R65 and GlyRα1^Q177G^ is possible. Characterization of G160 variants, localized in loop B, revealed that replacing glycine with two other small amino acids alanine and serine results in a 6- to 10-fold decrease in glycine potency (Atak et al., 2015). These data indicate that even the small difference between glycine and alanine can have a big impact on the conformation of the ligand-binding pocket. In contrast, characterization of P250 variants, a residue located in the short intracellular TM1-2 loop, has shown that short side chains gave rise to WT-like channels (Breitinger et al., 2001). Hence, the observed effects depend on the origin of the amino acid at a determined position in the protein.

The introduction of a cysteine at position 177 is also predicted to result in disturbed hydrogen network formation which is in line with a slight rightward shift in glycine EC_50_ values. Free cysteine residues share the ability for disulfide bridge formation. However, this is unlikely as C138-C152 and C198-C209 already form stable disulfide bridges in the GlyR (James et al., 2013). A disruption of those disulfide bridges would probably have stronger negative effects on ion channel function or even lead to non-functionality (Vogel et al., 2009). Sterically the effect of GlyRα1^Q177C^ is less detrimental which might explain the observed small effects on glycine potency. Tryptophan (W) is the largest amino acid and contains an aromatic indole ring. Because of the indole side chain, no hydrogen bond between GlyRα1^Q177W^ and R65 is possible. The original glutamine 177 forms a hydrogen bond with R65, which is located in the glycine binding pocket. Mutation of R65 to lysine and alanine revealed a 200- and 1250-fold decrease in glycine EC_50_, demonstrating that R65 is an important residue for ligand binding (Grudzinska et al., 2005).

The analysis of partial agonist efficacy and potency implies changes in agonist efficacy at least for taurine and changes in partial agonist potencies. These data indicate that Q177 influences the formation of the agonist/partial agonist binding sites possibly by differences in hydrogen bond network formation. Moreover, taurine measurements verified that structural changes in the β8–β9 loop also affect GlyR gating similar to findings for the *shaky* mouse mutant (Schaefer et al., 2017). In summary, the number of contacts to the hydrogen bond network and a mutual stabilization of the conformation seems to be reduced.

Since, the first description of the large movements the β8–β9 loop undergoes upon transition from the agonist-bound GlyR structure to the open conformation in the cryo-EM structure of GlyRα1 (Du et al., 2015), similar observations have been explored in the α4β2 crystal structure of the nicotinic acetylcholine receptor (Morales-Perez et al., 2016). The β8–β9 loop is connected to loop C covering the binding pocket but also is located underneath the binding pocket, thus it indirectly participates in the binding pocket. Furthermore, it is involved in the coupling of conformational changes of the ECD following ligand binding to finally ion channel opening and closing. During GlyR ion channel opening, the C loop switches from open to closed and displaces the β8–β9 loop which concomitantly induces rotation of the pre-M1 and M1 and finally movement of the M2 elements (Du et al., 2015). The analysis of the first mouse mutant carrying an amino acid transition in the β8–β9 loop further revealed the importance of this loop structure for GlyR function and survival. The mutant mouse showed no changes in agonist affinity but the ensuing signal transduction resulting in ion channel opening was almost completely abolished (Schaefer et al., 2017). We showed that all mutations at residue Q177 lack the potential to form hydrogen bonds with residue R65. Thus, the hydrogen bond to R65 is critical for the stabilization of the glycine-binding pocket, which is in agreement with the observed decrease in glycine potencies. A further impact on GlyR gating as suggested by the observed reduced efficacy of the partial agonist taurine is in line with the displacement β8–β9 undergoes during GlyR ion channel opening and closing and glycinergic signaling toward the pore forming unit (Du et al., 2015).

Moreover, the β8–β9 loop harbors a structural potential for allosteric modulation. Nys et al. (2016) argued that chlorpromazine bound to ELIC, a member of the Cys-loop receptor family, undergoes hydrophobic interaction with β8–β9 loop residues and is part of a multisite model for allosteric modulation in a continuous stretch from the top to the bottom of the receptor. A modulatory role of the β8–β9 loop has also been determined for GABA_A_ receptors. Tricyclic oxazole-2,3-benzodiazepines bind to the interface between α and β subunits to residues ^172^REPAR^176^ within the GABA_A_ receptor β8–β9 loop. These residues undergo interactions with residues R66 and F45 located close to the agonist binding site (Mihalik et al., 2017). The β8–β9 of the GlyR and other Cys-loop receptors might also harbor allosteric potential for yet unknown modulators.

## Conclusion

The β8–β9 loop is an important contributor to the hydrogen bond network in the ligand-bound state thus stabilizing the glycine-binding pocket.

## Author Contributions

CV participated in research design. CV and DJ conducted experiments. CD, HS, CV, NS, and DJ performed data analysis. CV and NS wrote the manuscript.

## Conflict of Interest Statement

The authors declare that the research was conducted in the absence of any commercial or financial relationships that could be construed as a potential conflict of interest.

## Footnotes

1 https://imagej.net/ImageJ

2 https://imagej.net/ImageJ

3 http://www.ebi.ac.uk/Tools/msa/clustalo/

## Abbreviations

ECD: extracellular domain
GlyR: glycine receptor
TMs: transmembrane domains
WT: wild type
